# Perspectives of pteridophytes microbiome for bioremediation in agricultural applications

**DOI:** 10.1515/biol-2022-0870

**Published:** 2024-05-31

**Authors:** Yasaswinee Rout, Soumya Sephalika Swain, Madhusmita Ghana, Debabrata Dash, Shubhransu Nayak

**Affiliations:** Central National Herbarium, Botanical Survey of India, 711103, Howrah, West Bengal, India; Odisha Biodiversity Board, Nayapalli, Bhubaneswar, 751015, India

**Keywords:** agriculture, bioremediation, heavy metal, pteridophyte, microbiome

## Abstract

The microbiome is the synchronised congregation of millions of microbial cells in a particular ecosystem. The rhizospheric, phyllospheric, and endospheric microbial diversity of lower groups of plants like pteridophytes, which includes the Ferns and Fern Allies, have also given numerous alternative opportunities to achieve greener and sustainable agriculture. The broad-spectrum bioactivities of these microorganisms, including bioremediation of heavy metals (HMs) in contaminated soil, have been drawing the attention of agricultural researchers for the preparation of bioformulations for applications in climate-resilient and versatile agricultural production systems. Pteridophytes have an enormous capacity to absorb HMs from the soil. However, their direct application in the agricultural field for HM absorption seems infeasible. At the same time, utilisation of Pteridophyte-associated microbes having the capacity for bioremediation have been evaluated and can revolutionise agriculture in mining and mineral-rich areas. In spite of the great potential, this group of microbiomes has been less studied. Under these facts, this prospective review was carried out to summarise the basic and applied research on the potential of Pteridophyte microbiomes for soil bioremediation and other agricultural applications globally. Gaps have also been indicated to present scopes for future research programmes.

## 1 Introduction

Industrialisation, agricultural intensification, waste disposal, and its associated activities have directed the production and release of several chemical compounds such as organic solvents, chemical fertilisers, pesticides, pigments, dyes, and plastics to the surroundings, which has contributed to the gross degradation of the environment. Aside from all of these environmental contaminants, heavy metals (HMs) are considered to be one of the most significant noxious elements that are harmful to agricultural crops and humans subsequently. In addition to these factors, diesel is one of the most frequently reported causes of soil and water contamination due to extensive transportation and application in automobiles and industrial sectors. Parallel to this, scientists are developing greener farming practices in order to replace synthetic chemicals in use and enhance soil health through bioremediation. These alternatives usually come from plants, animals, and microbial resources. Plants and soil harbour millions of microorganisms, which collectively form a microbial community known as the “microbiome” that imparts a number of beneficial effects on the plants and ecosystem [1,2]. Microbial strains from various sources or microbiomes have been utilised in agricultural operations as an option for eco-friendly and sustainable agriculture production systems. The application of an efficient and diverse soil microbiome backed by modern technologies can facilitate and promote sustainable agriculture and can effectively contribute to meeting the triple requirements of economic, social, and environmental sustainability. Soil, plants, and special habitats like hot springs, forests, and contaminated areas have been primarily used as sources of these beneficial microbes and microbiomes.

However, microbiomes associated with lower plants like “Pteridophytes,” which include Ferns and Fern allies, have also attracted the attention of the scientific communities as sources of novel bioactive secondary metabolites [3]. Ferns are one of the most significant plant groups because of their greater diversity, especially in tropical areas. Beneficial microbes are abundantly associated in the rhizosphere, phyllosphere, and endosphere of various species of ferns. Ferns are known to possess the capacity for removing contaminants *via* accumulation, chelation, and detoxification mechanisms, and this property helps them to survive in stressed ecosystems like mineral-rich soils [4,5,6]. The fibrous root system supports pollutant degradation by providing favourable environmental conditions for microbial activities and pollutant metabolism [7]. Various studies have proved that microbes associated with ferns absorb HMs through their cells and also enhance the bioaccumulation of minerals in plant biomass, which makes them ideal candidates for soil bioremediation [8,9,10].

In spite of this great potential, fern-associated microbes have been less studied regarding their practical applicability for bioremediation of agricultural soil. To date, no formulation has been developed globally with microbial strains isolated from ferns. Usually, culturable microorganisms could be used more feasibly for practical applications than their genome in the environment. Hence, more exploration of microbial diversity is required in various species of ferns from different habitats to generate more insights into the fern–microbe interactions. Under these facts, the current review was carried out to discuss the enormous potential of various microbial groups associated with Pteridophyte (ferns and fern allies) regarding soil bioremediation and to put light on research gaps of utilisation in agriculture.

## 2 Methodology for data collection and systematic literature review

**Figure 1:**
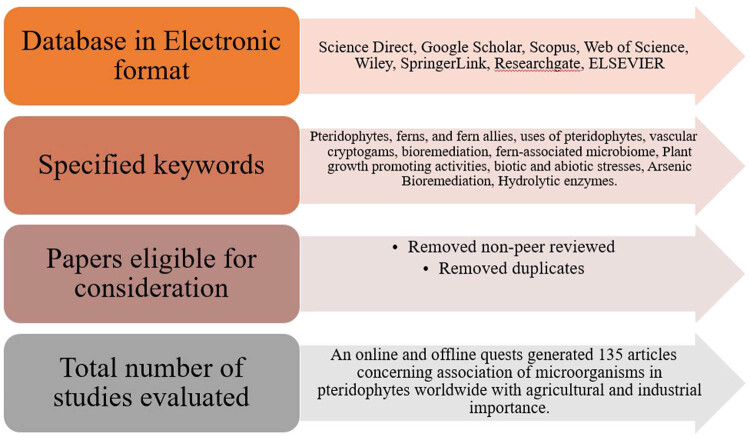
Structural outline of the article selection process for a systematic article review.

Global literature on the microbial significance of pteridophytes was collected from scientific journals (local, regional, and global), books, book chapters, magazines, conference/seminar proceedings, unpublished/published M.Sc. and Ph.D. dissertations, and other online databases, including Research gate, PubMed, Science Direct, DOAJ, Google Scholar, and Web of Science. To ensure a comprehensive and thorough study, a systematic review methodology has been used. This included collection, evaluation, and synthesis of evidence from a variety of sources [11,12]. This approach included searching databases, screening titles and abstracts, and then critically appraising the studies in order to assess their validity and reliability. Systematic reviews provide a comprehensive analysis of published and unpublished evidence on a given research topic by which it is easy to identify, assess, and synthesise the findings of all relevant studies [13]. While this study focuses on the importance of microorganisms associated with pteridophytes from the perspective of agriculture and HM tolerance capacities among other plants worldwide, the acquired results from search engines are correlated with the review’s objectives. The steps of a systematic review are presented in Figure 1.

## 3 Results and discussion

### 3.1 Ecological significance of pteridophytes: the “Plant Amphibians”

Pteridophytes or Vascular cryptogams or ferns and fern allies are also termed as “Amphibians” of the Plant Kingdom since they need water for fertilisation. They consist of non-flowering and primitive vascular plants that occur in humid and cooler localities [14]. These are an ancient group of plants which, according to the Five Kingdoms classification, include the Filicinophyta (ferns), Sphenophyta (horsetails), Lycophyta (lycopods), and Psilophyta (whisk ferns). They are represented by between 12,000 and 15,000 species and are most widely distributed in the tropics [15,16]. About 13,600 species belonging to 305 genera are found worldwide [17], out of which 1,200 species belonging to 70 families and 191 genera are found in India [18,19,20]. Pteridophytes can grow on trees (epiphytes), on crevices of rocks (lithophytes), and even found completely immersed in water (hydrophytes) [21].

**Figure 2:**
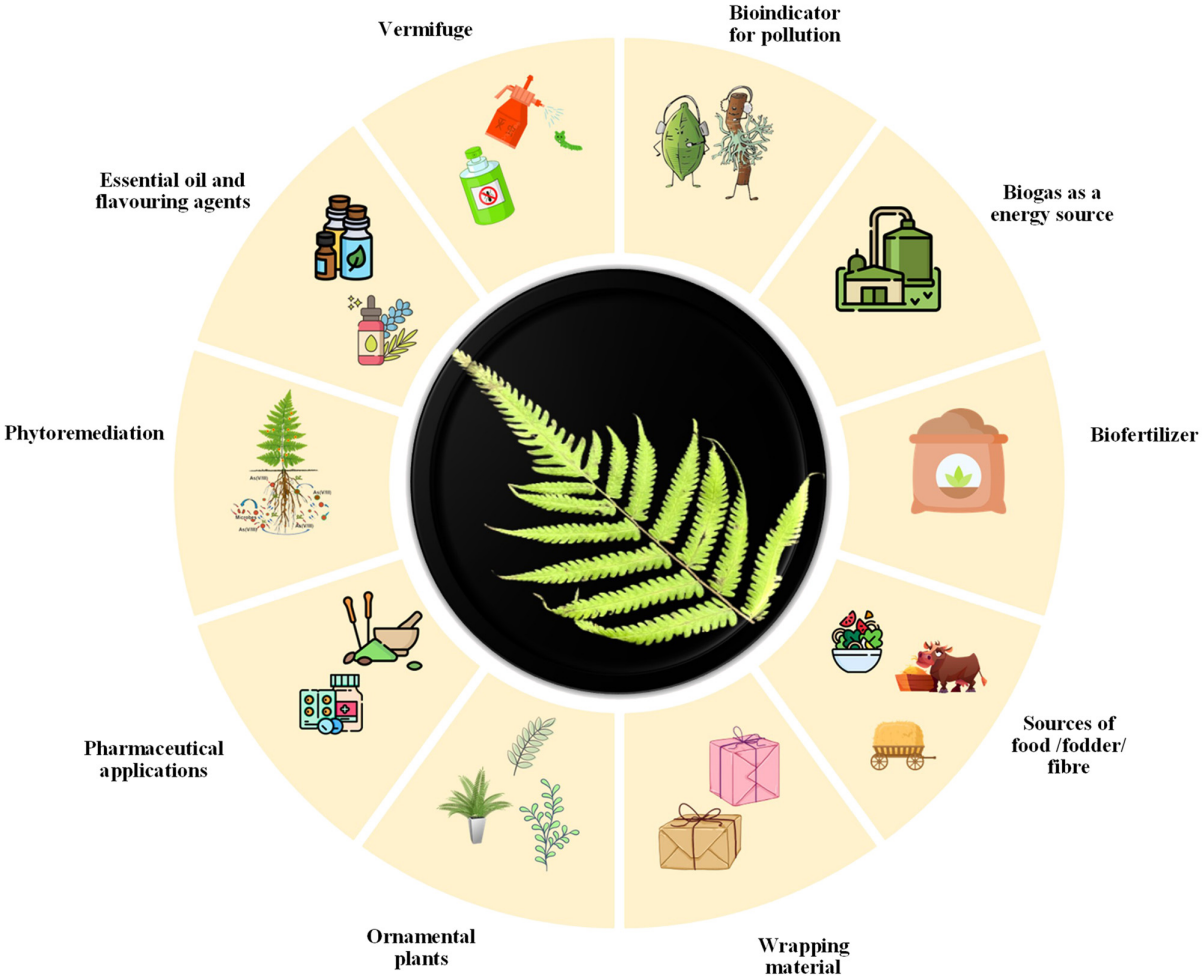
Environmentally important pteridophytes play a vital role in the ecosystem.

About 170 species of pteridophytes have been reported to play important roles in food, medicine, biogas, phytoremediation, biofertiliser, bio-indicator for pollution, etc. (Figure 2) [22]. Pteridophytes are not just randomly distributed in a locality, as the condition of their microhabitats is determined by soil texture, soil fertility, atmospheric conditions, precipitation, and light intensity [20,23]. They are considered excellent ecological indicators (EI) of various environmental factors such as soil type, environmental integrity, climate change, environmental pollution, association with other groups of organisms, etc. Ferns belonging to subclasses Polypodiidae, Equisetidae, Ophioglossidae, and Marattiidae of the family Polypodiopsida are cited as EIs. *Asplenium* sp., *Blechnum* sp., *Cyathea* sp., *Pteridium* sp., *Equisetum* sp., *Dicranopteris* sp., *Polypodium* sp., *Adiantum* sp., and *Pteris* sp. are some commonly cited EIs [24].

### 3.2 Microbial diversity associated with pteridophytes

**Figure 3:**
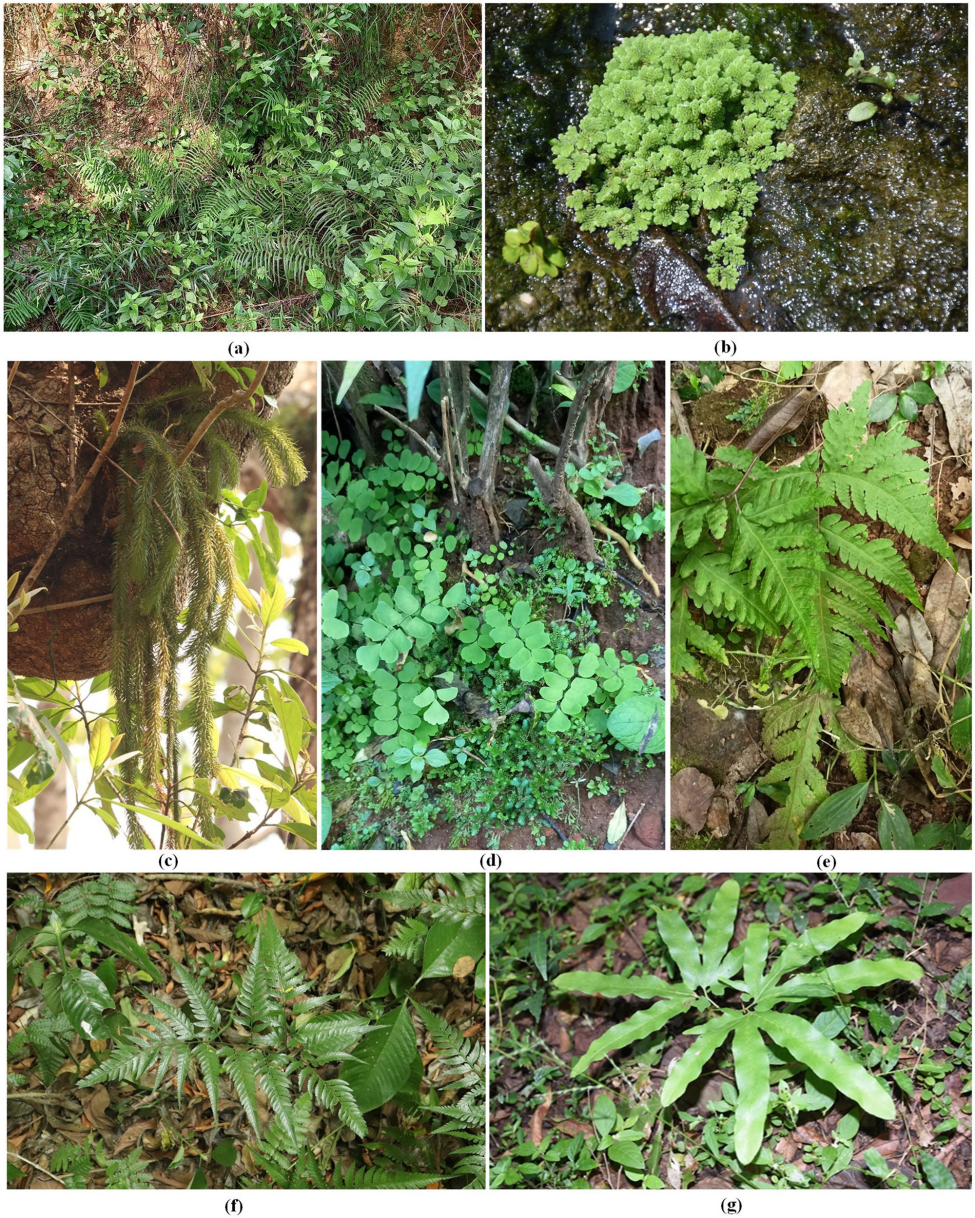
Predominant Pteridophyte species having microbial association with higher bioactivities for potential applications in agriculture: (a) *Pteris vittata*, (b) *Azolla* sp., (c) *Huperzia* sp., (d) *Adiantum* sp., (e) *Tectaria* sp., (f) *Dryopteris* sp., and (g) *Lygodium* sp.

Like any other terrestrial and aquatic plants, Pteridophytes are also found to be closely associated with a wide diversity of microorganisms, especially bacteria, fungi, actinomycetes, etc. (Figure 3). Numerous studies have been conducted by various researchers around the globe to explore and enumerate the pteridophyte-associated microbial diversity. They play vital roles in the health of both the host plant and the ecosystem, such as nutrient supply, resistance against biotic and abiotic stresses, production of growth hormones and secondary metabolites, etc. [25]. However, less research has been conducted regarding the applicability of those microorganisms and those to only specific subject areas. These microorganisms could exhibit a number of beneficial attributes not only to the host plant but also could be used independently as per their application potential. In particular, the fern-associated microbes showed greater bioactivity for potential use in agriculture. Mostly, bacterial species that have been isolated from many common ferns of specific habitats exhibited plant growth-promoting activities, inhibited plant pathogens, and imparted tolerance to salinity. Furthermore, these groups of bacteria also showed a higher potential to absorb and neutralise many HMs. However, there are still many species of ferns that remain to be explored, and activities like bioremediation need to be studied more extensively for the utilisation of these groups of microbes for application in agriculture in affected areas. Hence, it is very much essential to consolidate the relevant studies to date to enumerate the significance of fern-associated microbiomes regarding their potential to absorb HMs in biological soil remediation.

### 3.3 Application of fern-associated microorganisms for soil bioremediation

#### 3.3.1 Soil contamination with HMs

In general, the HMs having a density higher than 5 g/cm^3^ are non-biodegradable, toxic, or poisonous even in lower concentrations. The HMs and metalloids, including arsenic (As), chromium (Cr), manganese (Mn), cobalt (Co), nickel (Ni), zinc (Zn), copper (Cu), cadmium (Cd), tin (Sn), lead (Pb), mercury (Hg), and iron (Fe), can result in significant toxic impacts. Some of these metals are known as micronutrients and are required for plant growth, while others have unknown biological functions and are toxic even at low concentrations [26,27,28]. Studies have shown that the increase in HM content in wheat and corn plants near the mines that are under exploitation or abandoned, is higher than in the uncontaminated areas [29]. These contaminants can remain in the environment for decades or centuries, which increases the risk of accumulation of HMs in living organisms and soft tissues, which in turn affects the normal functioning of neurological, immune, gastrointestinal, and cardiovascular systems [30,31]. These HMs can disrupt the normal structure and function of cellular components and impede various metabolic and developmental processes, which ultimately have adverse effects on crop health and productivity [32]. From cultivated land to our food basket, metal contamination has a significant impact on agriculture. Profound exposure of living organisms to these HMs can have harmful effects, especially in the case of human beings. It may cause serious health issues like mental retardation, birth defects, autism, psychosis, and paralysis [33].

#### 3.3.2 Bioremediation of HMs

The bioremediation process is a highly promising, effective, and economical process that is mediated by living organisms, mainly microorganisms and green plants, and their enzymes to remove, degrade, mineralise, transform, and detoxify the hazardous pollutants from the contaminated environment into nontoxic or less toxic forms [34,35]. Even though angiosperms tend to dominate the phytoremediation process, ferns have now been utilised more and more recently as well [36].

Microorganisms are known to play a huge role in the biogeochemical cycle of metal transformations between soluble and insoluble forms [37]. Compared to animals and plants, microorganisms are known to have a greater resistance to environmental stress by transforming the toxic HMs into less toxic or nontoxic forms through metabolic pathways and utilise them for their growth [10]. Various strains of microbes from different sources, such as soil, water, sediments, and plants, have been used as a method of restoration of a contaminated environment. Even though the ability of pteridophytes to remove pollutants has been studied extensively, a lesser number of investigations have been carried out on the microorganisms associated with these groups of plants that could have equal or even more potential for remediation of soil HMs.

#### 3.3.3 Potential of pteridophyte microbiome for soil bioremediation

A notable example of ferns is *Pteris vittata,* which is known to grow in HM-contaminated soils like mining areas. This species is one of the most common hyperaccumulators of HMs [5,38], i.e. more than 3,000 mg kg^−1^ as above-ground biomass [38]. Other species belonging to the genus *Pteris* of order Pteridales are also known as arsenic hyperaccumulators. Apart from this, other ferns like *Adiantum capillus veneris* and *Pityrogramma calomelanos* also accumulate arsenic [5], while *Nephrolepis cordifolia* and *Hypolepis muelleri* are known as copper (Cu), lead (Pb), zinc (Zn) and nickel (Ni) phyto-stabilisers. Fern belonging to the genus *Adiantum* are known to be phytoextractors of lead (Pb) and nickel (Ni).

In recent years, fern-associated microorganisms have been utilised majorly to explore the potential for arsenic bioremediation. Most of the studies were confined to bacterial species isolated from the endosphere and rhizosphere of common ferns like *Pteris vittata* (Chinese Brake) and *Pteris multifida* (Spider Brake Fern). Bacterial isolates reported from these two plants are more or less similar and mostly belong to genera *Bacillus*, *Pseudomonas*, *Brevundimonas*sp, *Rhizobium*, *Acinetobacter*, *Arthrobacter*, *Paenarthrobacter*, *Paeniglutamicibacter*, *Rhodococcus*, *Microbacterium, Flavobacterium*, *Sinorhizobium*, *Ochrobactrum*, *Cupriavidus*, *Serratia*, etc. Even studies have revealed 50 times more presence and 3 to 12 times more diversity of bacterial communities and their functional genes (*aroA*-like) genes in the rhizosphere of *P. vittata* on amendment of arsenic in the soil [39]. Das [40] reported an alteration of rhizospheric microbiomes of *P. vittata* upon As-enrichment. The study revealed a noticeable increase in the relative abundance of *Proteobacteria* (12.3%)*, Actinobacteria* (39.5%), and *Chloroflexi* (143%), whereas lowered *Bacteroidetes* (21.6%)*, Acidobacteria* (19%)*, Verrucomicrobia* (9.8%) and *Firmicutes* (5.6%) composition in the rhizospheric soil. A substantial increase in both soil enzyme activity involved in the carbon, nitrogen, and phosphorus cycle, and fond biomass, as well as an increase in gene abundance of As-transforming bacteria, Fe- and S-reducing bacteria, and N- and C-fixing bacteria, were reported in the rhizosphere of the plant after As-enrichment. All these microorganisms were formerly reported to play a significant role in As-oxidation and reduction.

Most of the isolates not only exhibited the capacity to degrade toxic HMs but also possessed different plant growth-promoting (PGP) attributes, such as the production of IAA, siderophores, P-solubilisation, and ACC deaminase activity [41,42]. Other than arsenic, microbes associated with *Pteris vittata* also possessed the potential of bioremediation of other HMs like nickel (Ni), vanadium(v), chromium (Cr), and lead (Pb). These potential bacterial strains have been proven to be carrying genes encoding robust responses to HM stress.

### 3.4 Arsenic bioremediation

According to World Health Organisation reports, at least 140 million people globally are exposed to arsenic at levels exceeding the limits of the provisional guideline values [43]. Arsenic has been declared as the most prevalent hazardous substance in the environment and is classified as a Group 1 carcinogen. Because of its chronic and widespread impacts, there has been a renewed interest in arsenic as a pollutant issue, which has led the US Centres for Disease Control and Prevention to place it at the top of the ATSDR’s Substance Priority List [44,45]. A massive mass poisoning of a population in history, as defined by the World Health Organisation, occurred in Bangladesh, where an estimated 125 million people were exposed to inorganic arsenic from polluted tube wells, causing arsenic to become globally notorious [46]. Recently, in countries like India, China, Argentina and USA, arsenic (As) contamination has become a growing concern for public health [47,48].

Natural arsenic occurs in four oxidation states: arsenate (As(v)), arsenite (As(iii)), elemental arsenic (As(0)), and arsenide (As^−III^). When combined with other elements, it forms organic and inorganic arsenic. Inorganic compounds are considered to be more toxic than the organic ones. Further, trivalent methylated arsenic has been found to be more dangerous for humans since it can break down DNA more efficiently [49,50].

Microbial diversity associated with Pteridophytes could be exploited as an inexpensive and environmentally friendly tool to reduce arsenic (As) contamination. A study by Han et al. [39,51] revealed that both rhizospheric and endophytic bacteria of *Pteris vittata* played a significant role in As-transformation. Out of 18 species isolated from tissue extracts of this fern, 9 species belonged to Proteobacteria, 8 Firmicutes, and 1 Bacteroidetes. Rhizobacteria is dominated by phylum *Proteobacteria,* while 62% of endobacteria belong to Firmicutes. Further investigation showed that As(v) reduction was only found in rhizome and fond extracts at 3.7–24% of As v, whereas 45 and 73% As(iii) oxidation was found in root extract, indicating the role of both Rhizobacteria and endobacteria. Demonstrating the role of rhizospheric bacteria in arsenic bioremediation, Wang et al. [8] cultivated *Pteris vittata* plants (4–5 fonds) for 2 weeks in hogland solution along with 1 mg L^−1^ As(iii) in a hydroponic system. Bacterial strains belonging to Acinetobacter sp., *Comamonas* sp. *Flavobacterium* sp., *Staphylococcus* sp., and *Pseudomonas* sp. were found to be responsible for both As(iii) oxidation and As(v) reduction. Liu et al. [29] reported the potential of catecholate siderophore-producing *Pseudomonas* sp., previously reported from the rhizosphere of *Pteris vittata* [52], in bioremediation of Arsenic. The siderophore was found to be effective not only in the dissolution of FeSO_4_ but also increased the biomass of the plant.

Xu et al. [53] isolated 43 As-resistant bacterial endophytes from *Pteris vittata,* mostly dominated by *Proteobacteria* (47%), followed by *Actinobacteria* (42%)*, Bacteroidetes* (9.3%), and *Firmicutes* (2.3%). These endophytes mainly consisted of *Brevundomonas* sp.*, Rhodococcus* sp., *Microbacterium* sp., and *Flavobacterium* sp. Among them, six endophytes showed greater growth in the presence of 10 mM of As. Root endophytes were found to be more resistant to As(v), while leaflet endophytes were more tolerant to As(iii). Resistance of bacterial strains to As showed a positive correlation with reduction of As(v). Similarly, Tiwari [54] isolated eight different bacterial root endophytes from *Pteris vittata* belonging to three families: Proteobacteria, Firmicutes, and Bacteriodetes. Based on a preliminary test, only one isolate demonstrated As tolerance of up to 1,000 mg L^−1^. The *aox* gene was observed in two endophytes, indicating As(iii) oxidisation ability, whereas *arsB* gene was found in six isolates. All the isolates showed higher As-tolerance with a minimum inhibitory concentration ranging from 50 to 1,000 mg L^−1^.

Gu et al. [55] isolated 116 arsenite-resistant endophytic bacteria from roots of *P. vittata* with different As concentrations. Based on the 16S rRNA gene sequence analysis, the isolates were categorised into *Proteobacteria, Actinobacteria*, and *Firmicutes,* mostly dominated by genera *Agrobacterium, Stenotrophomonas*, *Pseudomonas, Rhodococcus*, and *Bacillus*. The most highly arsenite-resistant bacteria (minimum inhibitory concentration > 45 mM) were isolated from *P. vittata* with high arsenic concentrations and belonged to the genera *Agrobacterium* and *Bacillus*. The strains with high As tolerance also showed high levels of indole-3-acetic acid (IAA) production and carried arsB/ACR3(2) genes. The arsB and ACR3 (2) were most likely horizontally transferred among the strains.

The rhizospheric bacteria *Pseudomonas vancouverensis* strain m318 isolated from *Pteris multifida* contains *aio-A* genes and demonstrated high chemotactic responses as well as colonisation efficiency on roots from *P. vittata*, suggesting its broad host preferences. As-hyperaccumulation was significantly increased in *P. vittata* (48–146%) and *P. multifida* (42–233%) upon inoculation with the strain in field trials [56]. Abou-Shanab [57] evaluated the diversity of rhizospheric bacteria in *P. vittate* and their interaction in As-contaminated soil. A total of 44 of As-resistant endophytic bacteria were reported belonging to genera *Pseudomonas* sp., *Agrobacterium* sp., *Paeniglutamicibacter* sp., *Rhizobium* sp., *Bacillus* sp., *Sinorhizobium* sp., *Ochrobactrum* sp., *Rhodococcus* sp., *Cupriavidus* sp., *Arthrobacter* sp., *Paeniarthrobacter* sp., and *Paenibacillus* sp. of phyla *Proteobacteria, Actinobacteria,* and *Firmicutes.* The majority of these bacteria were resistant to As(v) rather than As(iii). Agnihotri [58] reported the *Bacillus cereus* strain from the rhizosphere of *Azolla microphylla.* The strain showed extreme tolerance towards As(v) (2,000 mg L^−1^) due to the presence of arsC gene, which confirmed the presence of functional *ars* operon in imparting arsenic resistance (Table 1).

**Table 1:** Bacterial diversity of ferns and their potential for As bioremediation

Bacterial species	Source plant and environment	Potential arsenic bioremediation activity	Experiment type	Reference
*Pseudomonas* sp., *Comamonas* sp,. and *Stenotrophomonas* sp.	Rhizospheric soil of *Pteris vittata*	Production of Siderophores and root exudates Enhanced plant As-uptake Increase in P uptake by *P. vittata*		[52]
*Bacillus* sp. and *Paenibacillus* sp.	Endophytes of *Pteris vittata*	Production of Siderophores and IAA Isolates from *P. vittata* and *P. multifida* showed higher tolerance to As(v) and As(iii), respectively Average IAA productions by isolates from *P. multifida* were higher than *P. vittata.*	Green house experiment	[59]
*Bacillus* sp., *Paenibacillus* sp., *Lysinibacillus* sp., *Massilia* sp., *Micrococcus* sp., *Brevundimonas* sp., *Paracoccus* sp., *Curtobacterium* sp., *Roseomonas* sp., *Staphylococcus* sp., *Sphingomonas* sp., and *Microbacterium* sp.,	Endophytes of *Pteris multifida*			
*Citrobacter* sp.	Endophyte of *Pteris vittata*	Bacterial isolates exhibited maximum resistance to As (400 mg L^−1^) and able to oxidize As(iii) and reduce As(v)	Pot culture	[60]
*Cupriavidus basilensis*	Rhizobacteria of *Pteris vittata*	Arsenic tolerance, rapid arsenite oxidation ability Accumulation of As up to 171% in *Pteris vittate*	Field trial near a Gold mine	[61]
*Bacillus* sp., *Paenarthrobacter ureafaciens,* and *Beijerinckia fluminensis.*	Endophyte of *Pteris vittata*	ACC deaminase activity, Siderophore, and IAA production	Green house trial	[62]

#### 3.4.1 Various mechanisms for arsenic remediation by microorganisms

**Figure 4:**
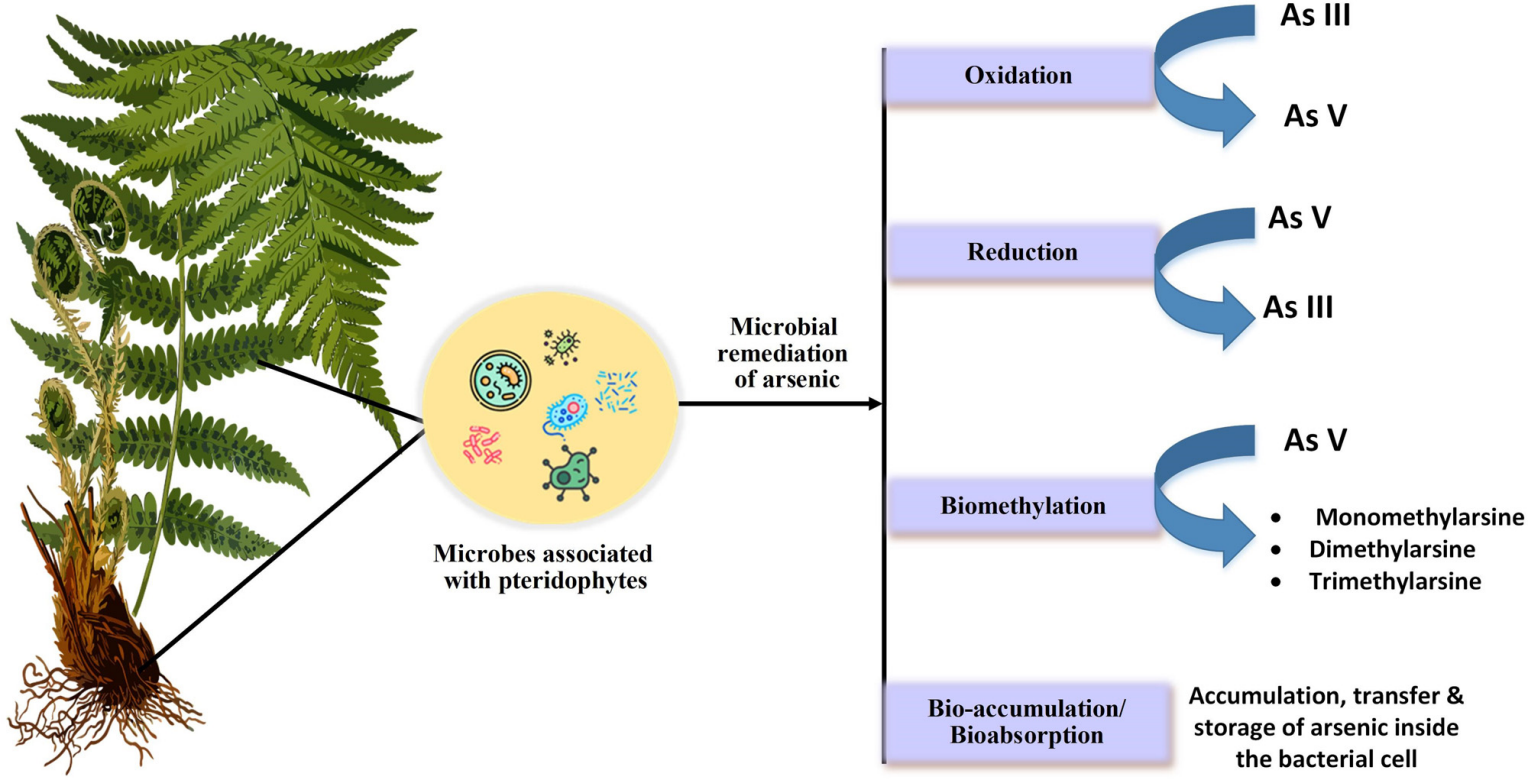
Divergent arsenic detoxification mechanisms used by microorganisms.

Microorganisms have adopted several mechanisms for survival under arsenic stress [63,64]. They combat the toxicity of arsenic through intrinsic properties or by using detoxifying mechanisms to survive. Numerous studies have been conducted to investigate these mechanisms underlying the bioremediation process, which includes cytosolic binding, efflux, precipitation, enzymatic and non-enzymatic reduction, and biofilm development. The arsenic biogeochemical cycle is highly dependent upon microbial transformation, which includes specific biochemical pathways (Figure 4) [65,66]. Arsenic accumulation, oxidation, reduction, volatilisation, etc., are different approaches practised by these microbes to resist the metalloid.

Microbial remediation of arsenic-contaminated soil has proved to be effective, reliable, and sustainable. The degradation of arsenic is a generating process that microbes use for their growth. Arsenite oxidation and arsenate reduction are the most effective methods. Bacterial arsenite is required for the bacterial arsenic oxidation pathway to activate, making it an inducible system [67]. The oxidation of arsenite (As(iii)) to form arsenate (As(v)) is considered an effective detoxification mechanism, which requires a periplasmic soluble enzyme, “arsenite oxidase” [68,69]. Investigation on arsenite oxidation, which is responsible for the detoxification process, reveals the existence of the aox (arsenite oxidising) operon containing the structural (aox A, aox B) and regulatory (aox R, aox S) genes [70]. Similarly, As(v) can also be reduced to As(iii) via two mechanisms, i.e. cytoplasmic arsenate reduction system and dissimilatory As(v) reduction encoded in the arr gene system. Microbial methylation is another multistep arsenic detoxifying method which involves transformation of solid or aqueous inorganic arsenic into gaseous arsines, i.e. arsine (AsH_3_), monomethylarsine (MeAsH_2_), dimethylarsine (Me_2_AsH), and trimethylarsine [71,72] through reduction of As(v), followed by oxidative addition of methyl group [73–75]. This process is catalysed by the enzyme As(iii) S-adenosylmethionine methyltransferase in microbes, which is encoded by the arsM genes [76]. The methylation process to mitigate arsenic contamination is under debate since the microbes responsible for the volatilisation of arsines only account for 0.5% of the microbial community [77,78]. However, the byproducts of the process still contain some level of toxicity; thus, many scientists do not consider this as a complete detoxification method [79]. Except these, bioaccumulation and bio-adsorption are also highly adapted by microbes, mostly bacteria, for arsenic removal. Although numerous studies are still being carried out by researchers to explore the arsenic-degradation capacity of the microbes, there is still a gap in knowledge on how to tap the capabilities of these essential microbes to their full potential.

### 3.5 Bioremediation of other HMs

*Serratia marcescens ss marcescens* PRE01, an endophyte of hyper-accumulator *P. vittata,* was reported to possess a significant ability to resist vanadium (1,500 mg L^−1^), chromium, and cadmium pollutants through the strong V(v) and Cr(vi) reduction and Cd(ii) adsorption. Apart from ACC deaminase activity and P solubilisation (336.41 mg L^−1^), the strain also exhibited production of IAA (60.14 mg L^−1^) and siderophore [80]. *Pseudomonas* spp. PG-12, an efficient rhizospheric bacterial strain isolated from *P. vittata*, was found to be effective in the bioremediation of HMs in contaminated soil due to its ability to resist multiple metals including cadmium (0.6 mM) and lead (10 mM) combined with the production of plant hormones such as indole acetic acid (IAA) (17.4 µg mL^−1^), gibberellins (3.54 µg mL^−1^). Genomic DNA analysis of the strain has identified metal efflux transporters such as PbrA, CadA2, and CzcR, which are likely to play a role in its metal resistance and detoxification. Sporophytes inoculated with the strain showed improved growth, increased P uptake, and reduced Pb uptake to plant tissue [81]. Banach [82] reported higher microbial diversity in Pb, Cd, Cr, Ni, Au, and Ag-treated *Azolla filiculoides,* an aquatic fern, than in the non-treated plant. The Pb, Cd, and Cr-treated plants were dominated by Cyanobacteria and Proteobacteria, followed by Actinobacteria, Firmicutes, and Bacteroidetes. In contrast, the core microbiome consists of *Acinetobacter, Asticcacaulis, Anabaena, Bacillus, Brevundimonas, Burkholderia, Dyella, Methyloversatilis, Rhizobium,* and *Staphylococcus* sp.

Yongpisanphop and Babel [83] reported five different endophytic bacteria from three different plants, including one metalliferous lead excluder fern, *Pityrogramma calomelanos*. Two endophytic bacteria belonging to genera *Pseudomonas* (*P. psychrophile* and *P. veronii*) were reported from the plant, among which *P. veronii* showed significant lead absorption potential in comparison to *P. psychrophile,* whereas both the organisms showed PGP traits as siderophore production and p solublisation activity.

Arbuscular mycorrhizal fungi have been commonly reported from the metal-contaminated sites [84,85]. The mycorrhizal symbiosis is known to help the host plants in nutrient and water uptake, thus enabling the plants in the establishment and survival of the host plant in different environmental niches. A number of studies reported the colonisation of mycorrhizal grasses from polluted sites [86] indicated the metal tolerance ability of AM fungi. Wu [87] reported 22 AM species from the rhizosphere of *Pteris vittate* collected from the Pb/Zn and As mining sites of China, mostly dominated by the genus *Glomus* like *G. brohultii, G. mosseae, G. microaggregatum*, and *G. geosporum*. Overall, the AM colonization of *P. vittate* collected from uncontaminated sites was found to be higher than the contaminated sites.

### 3.6 Bioremediation of oil/diesel contamination

Cohen et al. [88] conducted a field experiment to investigate diesel remediation by using aquatic fern *Azolla pinnata, Pistia stratiotes,* and *Salvinia molesta.* During the experiment, the bacterial colony that appeared after the death of the plant was reported to have dense growth in a 4% diesel-containing mineral salts medium and was found to lower the fluorescence from aromatic compounds by approximately 50% after 19 days. Similarly, Somiari et al. [89] reported bacterial endophytes *Pseudomonas* sp., *Bacillus* sp., *Klebsiella* sp.*, Nitrobacter* sp*., Staphylococcus* sp*., Nitrosomonas* sp*., Azotobacter* sp., and *Micrococcus* sp., isolated from roots of three mangrove plants including golden leather fern, i.e. *Acrostichum aureum* were capable of degrading crude oil. Out of these, the former two demonstrated the highest potential, i.e. 70 and 52.5%.

Though Pteridophyte-associated microbes have a great potential for soil bioremediation, there is still a huge gap in research regarding their practical utilisation. The bacterial isolates belonging to *Proteobacteria, Actinobacteria, Firmicutes, Bacteroidetes* were also formerly reported as active HM accumulators [90–93]. Hence, more studies need to be carried out on this group of bacteria in association with pteridophytes. In contrast, the potential of fungal diversity in these plants is still to be explored. The correlation between the plant growth hormone production (IAA) and bioremediation of HMs by microbes has also been reported in many studies [81], which indicates that these microorganisms not only accumulate toxic pollutants but also help the plants to thrive in adverse environments. The pathway and molecular interrelation among the two phenomena need to be studied more for better utilisation of these microbes.

### 3.7 Perspectives in the Odisha State of India

India is one of the 17 mega biodiverse countries of the world, known for its unique flora and fauna. The State of Odisha is situated in the eastern part of India and is one of the four maritime States bordering the Bay of Bengal. Due to its peculiar geographical locations, topography, and varied edaphic and climatic conditions, the biodiversity of Odisha is rich in terms of species richness. Physiographically, the state can be divided into four regions, viz., Northern Plateau, Eastern Ghats, Central Tableland, and Coastal Plains. According to Indian State Forest Report, 2021, the State of Odisha has a 33.5% forest coverage, which belongs to 19 forest types under 4 categories. Around 1,200 species of pteridophytes belonging to 70 families and 191 genera are found in India, whereas around 176 species are expected to occur in Odisha [94]. These are predominately distributed in forest areas that have mineral-rich soil (mining areas) and sufficient moisture. Besides these, many fern species also occur in wetlands, damp areas and coastal forests like mangroves in Bhitarkanika National Park. In spite of this vast biodiversity, to date, hardly any studies have been carried out in Odisha regarding the microbial association with pteridophytes. In India, few reports are available from the northeastern part only, and that is only restricted to some specific species. Most of these studies have focused more on diversity than their applicability. Results of preliminary experiments carried out by the authors of this article showed that both bacterial and fungal species, which have been isolated from pteridophytes existing in various forest-rich areas of Odisha, have tremendous potentials like antagonistic activity, enzyme production, and other traits for utilisation in agriculture. More exploration is underway for the identification of beneficial microbes and their utilisation in the composting of forest leaf litter and other biowastes.

## 4 Conclusions

Pteridophytes are still considered as understudied division under Kingdom Plantae. They hold a unique identity between the cryptogams and phanerogams and are considered critical drivers of succession. Although they are less conspicuous than other vascular plants, but they often contribute to the build-up of plant biomass and soil fertility. Since pteridophytes are host to numerous microorganisms that have potential for agricultural applications, extensive studies are required for a deep insight into their ability to decontaminate HMs from soil, which could be applied for agricultural purposes. Though metagenomics investigations have explored the genetic diversity of the fern microbiomes, a greater number of *in vitro* experiments need to be carried out and further translated into field applications. Since these lower plant groups are not preferred by herbivores, identification of potential pteridophytes and management of their associated microbial diversity could play a crucial role in the restoration of contaminated soil in the near future. This may open a path to greener ways of agriculture in areas with soil stress contaminated with HMs.
